# Changes in tibialis anterior architecture affect the amplitude of surface electromyograms

**DOI:** 10.1186/s12984-017-0291-5

**Published:** 2017-08-14

**Authors:** Taian M. Vieira, Maria Cristina Bisi, Rita Stagni, Alberto Botter

**Affiliations:** 1 Laboratory for Engineering of the Neuromuscular System (LISiN), Department of Electronics and Telecommunication, Politecnico di Torino, Via Cavalli 22/h, 10138 Torino, Italy; 2 Department of Electrical, Electronic and Information Engineering “Guglielmo Marconi”, University of Bologna, Viale Risorgimento 2, Bologna, 40136 Italy

**Keywords:** Electrical stimulation, Surface electromyograms, Ultrasound, Tibialis anterior

## Abstract

**Background:**

Variations in the amplitude of surface electromyograms (EMGs) are typically considered to advance inferences on the timing and degree of muscle activation in different circumstances. Surface EMGs are however affected by factors other than the muscle neural drive. In this study, we use electrical stimulation to investigate whether architectural changes in tibialis anterior (TA), a key muscle for balance and gait, affect the amplitude of surface EMGs.

**Methods:**

Current pulses (500 μs; 2 pps) were applied to the fibular nerve of ten participants, with the ankle at neutral, full dorsi and full plantar flexion positions. Ultrasound images were collected to quantify changes in TA architecture with changes in foot position. The peak-to-peak amplitude of differential M waves, detected with a grid of surface electrodes (16 × 4 electrodes; 10 mm inter-electrode distance), was considered to assess the effect of changes in TA architecture on the surface recordings.

**Results:**

On average, both TA pennation angle and width increased by respectively 7 deg. and 9 mm when the foot moved from plantar to dorsiflexion (*P* < 0.02). M-wave amplitudes changed significantly with ankle position. M waves elicited in dorsiflexion and neutral positions were ~25% greater than those obtained during plantar flexion, regardless of where they were detected in the grid (*P* < 0.001). This figure increased to ~50% when considering bipolar M waves.

**Conclusions:**

Findings reported here indicate the changes in EMG amplitude observed during dynamic contractions, especially when changes in TA architecture are expected (e.g., during gait), may not be exclusively conceived as variations in TA activation.

## Background

Surface electromyograms (EMGs) provide key, relevant information on the timing and relative degree of muscle activation [1, 2]. Temporal variations in the amplitude of surface EMGs, for instance, have been shown to successfully distinguish the modulation of calf muscles’ activity between different pathological gaits [3]. The possibility of non-invasively assessing the activity of ankle dorsiflexors [4, 5] covers specific clinical interest, due to its relevance for balance and locomotion. Given the tibialis anterior (TA) is a chief, ankle dorsiflexor, previous research has focused on the detection of surface EMGs to gain insights into the activation of TA in different populations and circumstances [6–8]. A general assumption of these previous studies is that variations in the amplitude of surface EMGs reflect variations in the relative degree of TA activation.

Changes in the amplitude of surface EMGs may not however indicate variations in the muscle neural drive. It is well established the amplitude of EMGs detected by surface electrodes positioned symmetrically at both sides of the muscle innervation zone may be remarkably small (i.e., end-plates’ location; [9, 10]). Similarly, thicker subcutaneous tissues lead to the detection of surface EMGs with smaller amplitude [11, 12]. Even though not well explored in the literature, changes in muscle architecture seem to constitute an additional, potential source of spurious variation in the amplitude of surface EMGs. Incipient accounts have reported indeed a significant effect of pennation angle on the amplitude of gastrocnemius, surface EMG; greater pennation angles result in more spatially localised and greater surface EMGs [13]. Whether or how much changes in TA architecture affect the distribution of myoelectric activity on the skin remains unknown. If changes in TA architecture lead to substantial changes in the amplitude distribution of surface EMGs, inferences on changes in the timing and degree of TA activity may therefore not be drawn from surface EMGs detected during conditions imposing changes in TA architecture (e.g., during dynamic contractions [14]).

Here we use nerve electrical stimulation and ultrasound images to investigate whether TA architectural changes affect EMGs detected by a grid of electrodes and by the conventional, bipolar montage. If architectural changes affect the amplitude of surface EMGs [13], then, current pulses applied to the fibular nerve with the ankle in different positions are expected to elicit different TA surface potentials (M waves). Otherwise, changes in muscle shape affect marginally the surface EMGs and therefore no variations in TA M waves are expected with changes in ankle position. To our knowledge this is the first study systematically reporting anatomically-induced changes in surface EMGs, of crucial relevance for the interpretation of TA myoelectric activity detected in dynamic contractions (e.g., during gait).

## Methods

### Participants

Ten healthy subjects (five women: 24–28 years; 53–60 kg; 152–170 cm; five men: 24–34 years; 72–96 kg; 178–188 cm) participated in this study, after providing written informed consent. Participants did not report any neuromuscular disorder during experiments. Experimental procedures conformed to the *Declaration of Helsinki* and were approved by the Institutional Ethical Committee.

### Stimulation protocol

**Fig. 1:**
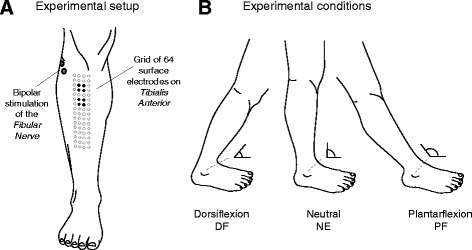
Electrodes’ and US probe positioning. **a**, shows the position of stimulation electrodes over the fibular nerve and of the 64 recording electrodes (8 × 4 arrangement) over the tibialis anterior (TA) muscle. See methods for detailed information on how the electrode grid was positioned over TA. Electrodes in the grid considered to simulate the conventional, bipolar detection are indicated with *black circles*. **b**, illustrates the three ankle joint positions for which the amplitude of M waves was quantified

Electrical stimulation was applied to the fibular nerve of participants’ right leg, while they stood upright and with their foot secured to a force meter (PY6, Bertec, USA). Monophasic current pulses (200us; Digitimer, UK) were delivered to the fibular nerve with pre-gelled, closely spaced electrodes (Fig. 1a; electrode size: 10 × 10 mm; 10 mm inter-electrode distance). Stimulation electrodes were positioned at the skin region closest to the fibular nerve, where the least injected current led to clearly observable, TA twitches.

Two sets of ten current pulses (2 pps) each were considered to elicit TA M waves, at 50% and 100% of the current intensity (range: 30–55 mA) over which no further increases in force could be observed. We decided to define the maximal current intensity based on force twitches rather than on M-wave amplitude because establishing the maximal M-wave amplitude from surface EMGs seems unviable for in-depth, pennate muscles [15]. Electrical stimulation was applied for three different, ankle joint angles. While standing with their right foot secured to the floor using straps, subjects were specifically asked to position their left foot (Fig. 1b): i) as much as possible backward (plantar flexion position; PF); ii) alongside the right foot (neural position; NE); iii) as much as possible forward (dorsiflexion; DF). The experimenter assisted subjects in changing position without losing balance and therefore without moving their right foot. A total of 60 stimulation pulses were applied per subject (10 pulses × 2 stimulation intensities × 3 ft positions). Stimulation order was randomised and 2 min rest was provided between stimulation sets.

### EMG, kinematic and ultrasound recordings

Monopolar EMGs were detected with a grid of 64 surface electrodes (16 × 4 electrodes; 8 mm diameter; 10 mm inter-electrode distance). Electrodes were secured to the skin with bi-adhesive foam and the electrode-skin contact was ensured by filling the foam cavities with conductive paste. The grid was positioned at a skin region covering as much as possible the TA superficial aponeurosis (Fig. 1a); the most medial column of electrodes was located 10 mm laterally to the tibial crest and the most proximal row was located 10 mm distally to the head of the fibula. Skin was cleaned with abrasive paste prior to placing the electrodes. EMGs were detected with a multi-channel fixed gain amplifier (W-EMG amplifier, LISiN-Politecnico di Toino, Italy; [16]). Trigger pulses indicating the onset of stimulation pulses were digitised synchronously with EMGs at 2441 Samples/s (24 bit A/D converter).

Ultrasound (US) images were recorded (Echoblaster 128, Telemed, LT) using a 4 cm linear array transducer (10 MHz) in both transverse and longitudinal planes [17]. The transducer was positioned tangentially to the skin, centred along the leg circumference defined by the fifth row of electrodes (Fig. 1a). Care was taken to ensure minimal pressure and thus to avoid distortion of image features related to the muscle tissue [17]. When recording transverse images, a series of contiguous images was sampled to provide a view of the entire muscle width. Longitudinal images were taken from the skin region between the second and third columns of electrodes, while ensuring fascicles could be clearly visualized from the deep to the superficial aponeurosis [18]. Images were collected for each of the three ankle positions, while subjects stood as shown in Fig. 1b. Subjects were instructed to relax their muscle as much as possible during both ultrasound and M-waves’ recording, otherwise changes in TA architecture resulting from changes in the degree of muscle contraction [19] would likely affect M waves. We ensured subjects held their muscle relaxed during electrical stimulation through visual inspection of surface EMGs; no single action potentials were observed during stimulation.

A cluster consisting of four reflective markers attached to the US probe was considered for calibration; 3D position of the US image plane in the cluster reference frame was calibrated based on previous reports [20, 21]. Eight reflective markers were attached on anatomical landmarks of the individuals’ right leg and foot (tibial tuberosity, head of the fibula, lateral malleolus, medial malleolus, first-, second-, fifth metatarsal and calcaneus) as indicated by Cappozzo and colleagues [22]. To ensure that the static ankle position was maintained during data acquisition and to track the cluster position of the US probe, kinematic data were collected at 100 Hz and offline synchronised via an external trigger.

### Data analysis

**Fig. 2:**
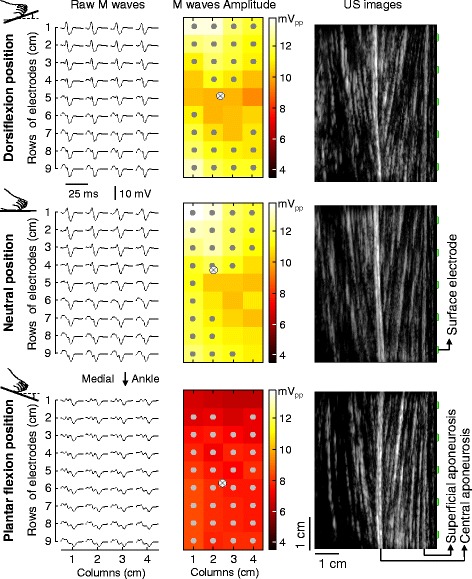
Electrically elicited responses, muscle architecture and ankle position. Raw, monopolar M waves (*left column*), their peak-to-peak amplitude (central column; greyscale images) and ultrasound images from TA (*right column*) are shown for a representative participant. *Grey circles* in the central column denote electrodes for which the M-waves peak-to-peak amplitude exceeded 70% of the maximal peak-to-peak amplitude obtained for each of the three ankle positions tested: dorsiflexion (*top row*), neutral (*middle row*) and plantar flexion (*bottom row*). Rectangles drawn in the right column schematically indicate the relative position between electrodes and TA fibres; note different rectangles are positioned over the superficial extremity of different TA fascicles

M waves detected with electrode grid: EMGs were first band-pass filtered with a fourth order, Butterworth filter (15–350 Hz cut-off frequencies). M waves were then obtained by averaging EMG samples over 30 ms epochs, with epochs starting from the onset of each trigger pulse; EMGs were averaged across pulses, separately for each electrode, stimulation intensity and ankle position (Fig. 2). The number of electrodes detecting relatively large M waves (termed *active channels*) and the region where these electrodes were located (the transverse and longitudinal coordinates of *active channels*’ centroid) were computed from both the M-waves peak-to-peak and root mean square amplitude. *Active channels* were identified by applying an automated technique to segment EMG images [23] and centroid coordinates were calculated as the weighted average of *active channels* across rows and columns (cf. grey and crossed circles in Fig. 2). Only electrodes located over the TA superficial aponeurosis were considered for analysis, given the amplitude distribution of EMGs detected in such region reflects the global degree of muscle activation [24].

M waves detected with bipolar electrodes: the effect of ankle position was tested on the peak-to-peak amplitude of M waves detected by the conventional, bipolar montage. Bipolar signals were simulated as the sample-by-sample difference between monopolar M waves averaged over two sets of four electrodes (Fig. 1a), centred in the location currently recommended for the positioning of bipolar electrodes on TA [25].

TA width and pennation: changes in TA width and pennation angle were estimated from US images. US images, acquired contiguously and transversally from TA, were projected in the 3D laboratory reference frame using the cluster probe pose and the previously performed calibration [20]; then, the images were superimposed and concatenated to provide a view of the entire muscle transverse section. Width was computed as the distance between the medial and lateral TA borders. Pennation angle was calculated from parasagittal images [26], as the acute angle between two intersecting lines representing fascicle orientation (line of the clearest fascicle) and deep aponeurosis [26].

Joint angles: Ankle angles were calculated from kinematic data, considering a 2-segment model of the right lower limb [27, 28]. After ensuring that static position was maintained during each test (standard deviation inferior to 1°), mean ankle angles were calculated for each subject and each position.

### Statistics

Parametric, inferential statistics were applied to test for the effect of ankle position on M-waves’ amplitude distribution and on muscle architecture characteristics, after ensuring homogeneity of variance (Levene’s test; W-values greater than 0.2) and Gaussian distribution (Shapiro-Wilk statistics *P* > 0.17) of both M-waves’ and architectural data. Two-way ANOVA was applied on M-waves’ amplitude distribution results, with ankle position as repeated measures (2 intensities × 3 positions). Pearson correlation analysis was applied to assess the association between M-wave amplitude and TA architecture. One-way ANOVA was applied on TA width and pennation angle, with ankle position as repeated measures (3 positions). Bonferroni correction was considered for post-hoc analysis.

## Results

### General considerations

Ankle DF corresponded to approximately 30° of flexion and PF to 40° of extension. Detailed pose data are shown in Table 1. When considering M waves, peak-to-peak and root mean square amplitude values provided statistically equivalent results. For convenience, we report results for the peak-to-peak amplitude.

**Table 1:** Median (5th and 95th percentiles) ankle angles estimated for the three foot positions

	Ankle angles		
Foot position	*FE*	*AA*	*IE*
DF	28 (12;43)	- 12 (−25;-5)	0 (−10;7)
NE	0 (−4;6)	-9 (−17;8)	9 (0;12)
PF	−37 (−41;-33)	1 (−4;8)	9 (2;15)

DF, NE and PF respectively correspond to dorsiflexion, neutral and plantar flexion

FE, AA and IE correspond respectively to Flexion/Extension, Ab/Adduction and Intra/Extra rotation

### Results from a representative participant

The effect of foot position on TA architecture and M waves is well appreciated by inspecting results from a single, representative participant. As shown in Fig. 2, the TA pennation angle increased from 5.5 to 13.0 deg. when this participant moved his right foot from PF to DF position (Fig. 2). Clearer M waves with greater amplitude were elicited with the foot in DF and NE rather than in PF position, regardless of where they were detected in the grid. Regional differences in M-wave amplitude were observed however, in particular for the DF position; in such position the similarity between M waves detected by different channels was less evident (cf. M waves and images in Fig. 2).

### Group results: Ankle position and M-wave amplitude distribution

**Fig. 3:**
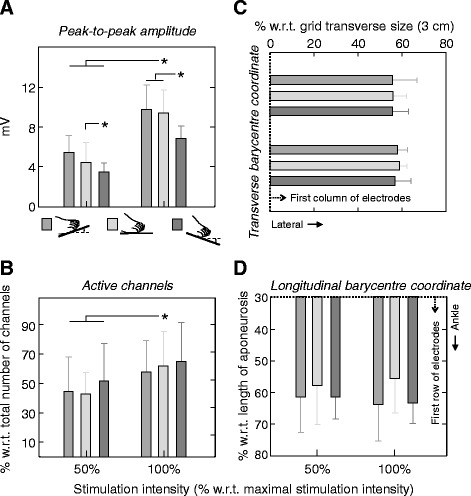
The spatial distribution of electrically elicited responses in tibialis anterior. Mean and standard deviation (whiskers; *N* = 10 subjects) are shown for the peak-to-peak amplitude of M waves (**a**), averaged across channels, the number of active channels (**b**) and the barycentre transverse (**c**) and longitudinal coordinates (**d**) within the grid. Different *grey* intensities indicate the three ankle positions tested: dorsiflexion (*grey*), neutral (*light grey*) and plantar flexion (*dark grey*). Values obtained for 50% and 100% of the maximal stimulation intensity are shown from top to bottom in panel **c** and from left to right in panels **a**, **b** and **d**. *Asterisks* denote statistical significance at 5%

When considering all participants, ankle position affected significantly M-waves’ peak-to-peak amplitude though not its distribution on the skin. M-waves’ amplitude was significantly smaller in PF than in NE and DF positions, both for 50% and 100% of the maximal stimulation intensity (Fig. 3a; ANOVA; *P* < 0.001). The size (i.e., number of *active channels*) and centre of M-waves’ amplitude distribution were however not affected by ankle position (*P* > 0.31), even though relatively larger peak-to-peak values tended to be detected over a larger and distal region in PF (Fig. 3b–d). Stimulation intensity, on the other hand, affected both the mean peak-to-peak amplitude (Fig. 3a) and the number of *active channels* (Fig. 3b; *P* < 0.038 in both cases), with smaller and more spatially diffused peak-to-peak values being obtained in PF position.

**Fig. 4:**
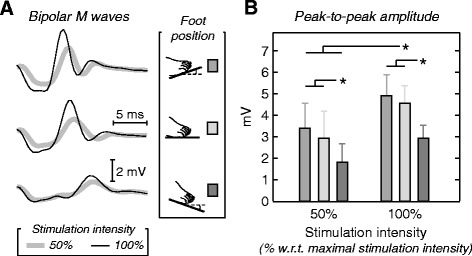
Effect of ankle position on bipolar M waves. Raw, bipolar M waves obtained for 50% (*thick, grey* traces) and 100% (*thin, black* traces) of the maximal stimulation amplitude are shown in **a** for a representative participant. From top to bottom, M waves were obtained with the foot at dorsiflexion, neutral and plantar flexion positions. **b**, shows mean and standard deviation (whiskers; *N* = 10 subjects) values for the peak-to-peak amplitude of bipolar M waves, with *grey* intensities indicating the three ankle positions tested: dorsiflexion (*grey*), neutral (*light grey*) and plantar flexion (*dark grey*). Values obtained for the 50% and 100% of the maximal stimulation intensity are shown from left to right. *Asterisks* denote statistical significance at *P* < 5%

As for monopolar signals, when moving the foot from dorsi to plantar flexion, the bipolar M waves became smaller and wider (cf. waveforms in Figs. 2 and 4a). When compared to DF and NE, the PF position provided M waves with significantly smaller peak-to-peak amplitude for the two stimulation intensities considered (Fig. 4b; *P* < 0.001). Moreover, for all ankle positions, peak-to-peak values of bipolar waves almost doubled from 50% to 100% stimulation intensity (*P* < 0.001).

### Muscle architectural changes with ankle position

Muscle architecture changed with foot position. As shown in Table 2, pennation angle and muscle width increased significantly with the ankle dorsiflexion.

**Table 2:** Average (standard deviation) values are reported for the pennation angle and for the width of the tibialis anterior muscle, both estimated for the three foot positions

	Tibialis anterior architecture	
Foot position	*Pennation angle (deg)* ^a^	*Muscle width (mm)* ^a^
DF	15.3 (3.7)	67.8 (8.0)
NE	10.7 (4.5)	59.8 (7.2)
PF	8.0 (1.7)	58.1 (5.0)

DF, NE and PF respectively correspond to dorsiflexion, neutral and plantar flexion

^a^indicates effect of foot position with *P* < 0.02

### The association between M-wave amplitude and muscle architecture

**Fig. 5:**
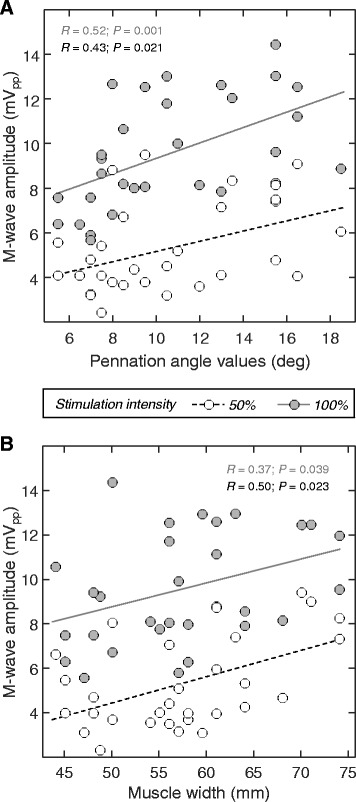
The association between changes in TA architecthre and M-waves amplitude. Scatter plots illustrate how the amplitude of M waves detected from TA changes with variations in the muscle pennation angle (**a**) and width (**b**). Data obtained for the three ankle joint angles are overlaid. The amplitude of M waves elicited for 50% and 100% stimulation intensitites are respectively represented with white and grey circles. Lines denote the regression lines calculated between M-waves amplitude and TA architecture measures. Pearson correlation coefficients and their *P* value are shown within the plots

The amplitude of M waves was significantly associated with both TA pennation angle and width. Greater pennation angle and width resulted in M waves with greater amplitude (Fig. 5), both during 100% (Pearson *R* > 0.36; *P* < 0.04; *N* = 30; 10 subjects × 3 ankle angles) and 50% stimulation intensities (Pearson *R >* 0.42; *P* < 0.025; *N* = 30). Similar, significant results were observed for bipolar M waves (Person *R* > 0.35 and *P* < 0.035 in all cases).

## Discussion

### Isolating the effects of changes in TA architecture on surface EMGs

Three considerations are necessary before interpreting current results. First, the effect of changes in muscle architecture on the amplitude of surface EMG was assessed during electrically elicited rather than during voluntary contractions. In the latter case, the sensitisation of joint, muscle and tendon receptors, resulting from changes in foot position, could lead to changes in muscle activity [29]. During voluntary contractions, it would thus not be possible to dissociate the effect of architectural changes from that of physiological mechanisms on the amplitude of TA M waves. Second, changes in TA architecture were induced by changing foot positioning while keeping the knee joint angle constant (Fig. 1a). We decided to keep the same leg-thigh orientation for the different ankle joint angles to ensure minimal, potentially negligible, changes in the relative position between the fibular nerve and stimulation electrodes. Even if the fibular nerve had moved in relation to the stimulation electrodes when moving the foot from DF to PF, these relative movements would be likely random and therefore would not explain the consistent changes in M-wave amplitude with foot positions across participants (Figs. 3, 4). Finally, TA M waves appear a few milliseconds after current pulses are delivered to the fibular nerve and last roughly 20 ms (cf. Fig. 2; [30]). Considering the force twitches develop within somewhat longer periods, the M waves obtained here are therefore unlikely affected by TA architectural changes associated with the force twitches. Collectively, these arguments suggest the results reported here are presumably not due to factors other than the change in TA architecture.

### Changes in TA architecture affect the amplitude of surface EMGs

The relationship between architectural changes and EMG amplitude is likely muscle dependent. For muscles with fibres oriented parallel to the skin, for example, it is well established that shifts in the innervation zone, resulting from changes in joint angle, affect the amplitude of surface EMGs; EMG amplitude is greatly underestimated when detected nearby the innervation zone [9, 10]. For in-depth pennate muscles, such as gastrocnemius and TA, the effect of changes in muscle architecture on the surface EMG is less well documented. Comparing current and previous results is therefore currently not possible. Recent, preliminary results, obtained with low intensity nerve stimulation, suggest however the amplitude distribution of surface EMGs changes with the gastrocnemius pennation angle [13]. For greater degrees of pennation, the projection of gastrocnemius fibres on the skin decreases, leading to the detection of greater EMGs at more localised and proximal gastrocnemius regions (cf. Fig. 4 in Mesin et al. [13]). Current results on TA partly corroborate these previous findings; we observed TA pennation affected M-wave amplitude though not its distribution (Figs. 2, 3, 4). Likely because we excited a somewhat great proportion of muscle fibres, changes in the distribution of M-wave amplitude with variation in foot position did not reach statistical significance (Fig. 3b–d). Additionally, differently from gastrocnemius, TA fibres are roughly symmetrically angled with respect to the muscle intermediate aponeurosis (Fig. 2; [31]). In virtue of the depth of the TA intermediate aponeurosis, action potentials of fibres running from the TA deep to intermediate aponeurosis would be expected to affect surface EMGs equally across electrodes (far-field potentials; [32]). Although we value the relevance of identifying the mechanisms underpinning the relationship between TA architecture and EMGs, here we were focused on understanding whether rather than how EMGs are affected by changes in TA architecture. Notwithstanding the potential causes, here we show that for a constant TA excitation surface EMGs with different amplitudes are detected for different foot positions.

Regardless of whether taken locally or not, EMG amplitude was affected by TA architectural changes. Given grids of electrodes sample from a somewhat large muscle region, they overtly provide surface EMGs less sensitive to spurious factors than the conventional, bipolar derivation [9, 15]. Here we show however the effect of TA architectural changes maybe not compensated for by sampling EMGs everywhere over the TA superficial aponeurosis; M-wave amplitude increased with the foot dorsiflexion for both recording modalities (Figs. 3a, 4b and 5). Therefore, surface EMGs should be interpreted carefully when collected from TA both with bipolar electrodes and with grids of electrodes.

### Practical implications for the interpretation of surface EMGs

Assumptions are often made whenever EMGs are used to advance considerations on the muscle function. Even though it is usually assumed the surface EMGs sample exclusively from the whole volume of target muscles, it has been shown the surface EMGs may sample from muscles other than that of interest (i.e. crosstalk; [30]) or, conversely, from a small, unrepresentative muscle region [15, 24]. In both cases, drawing inferences on the whole muscle level from single, localised EMG recordings maybe misleading. Of specific, practical interest here is the assumption that variations in the amplitude of surface EMGs indicate variations in the neural drive to the muscle; i.e., in the recruitment and firing rate of motor units. More specifically, increased and decreased amplitude of surface EMGs collected from a given muscle during a given condition are respectively conceived as augmented and diminished degree of muscle activity [4, 5]. The present study shows (Figs. 3 and 4) that this hypothesis is not always valid. Here we show that from 13% to 25% of the changes in the amplitude of TA M waves may be explained by changes in TA architecture (Fig. 5); even in the presence of a constant degree of excitation, M-wave amplitude was roughly 25% smaller in PF than in DF and NE positions (Fig. 3). When considering the conventional, bipolar montage, differences in M-wave amplitude with foot position increased to ~50% (Fig. 4). These results raise the question as to which extent variations in EMG amplitude observed in circumstances imposing TA architectural changes maybe attributable to physiological sources. In conclusion, while the possibility of compensating for these architectural effects on the EMGs urges further investigation, variations in EMG amplitude during dynamic conditions should be interpreted cautiously.

### Limitations

The generalisation of results to different muscles and circumstances is a key limitation of this study. Our decision to assess the TA muscle was motivated by its key role in human motion analysis [5, 26]. Although the variations in EMG amplitude with changes in foot position agree with previous observations for the gastrocnemius muscle [13], we refrain from stating current results generalise to in-depth pennate muscles other than TA. Anatomical differences between muscles (e.g. different degrees of pennation, or pennation in oblique planes) could lead to relationships between EMG and muscle architecture different from that reported here (Figs. 2, 3, 4, 5). Finally, it should be noted we may have underestimated the effect of TA architecture on M-wave amplitude. Considering the TA pennation angle increases with the degree of TA activity [14, 19], it is possible that M-waves’ amplitude would have increased to greater extents had we applied current pulses while subjects were sustaining a certain degree of TA activation. The caveat though is isolating the effect of TA architecture on the amplitude of M waves during voluntary contraction, as discussed above. While the generalisation of current results remains the subject of future investigations, it seems however likely that, for different muscles and conditions, changes in EMG amplitude result from structural in addition to physiological changes.

## Conclusions

In this study we used electrical stimulation to investigate whether the amplitude of surface EMGs is affected by changes in TA architecture. Architectural changes were elicited by changing foot position (Fig. 1). Key findings reveal (Fig. 3) the M-wave amplitude increased significantly as the foot moved from PF to DF position, regardless of where they were detected in the grid and of whether they were obtained with a grid or with bipolar electrodes. These findings indicate the changes in EMG amplitude observed during dynamic contractions, especially when changes in TA architecture are expected (e.g., during gait; [31]), may not be exclusively conceived as variations in TA activation.

## Abbreviations

DF: Ankle at dorsiflexion position
EMG: Electromyogram
NE: Ankle at neutral position
PF: Ankle at plantar flexion position
TA: Tibialis anterior

## References

[1] Nashner LM (1977). Fixed patterns of rapid postural responses among leg muscles during stance. Exp Brain Res.

[2] Nieuwenhuijzen PHJ (2007). A, Duysens J. Proactive and reactive mechanisms play a role in stepping on inverting surfaces during gait. J Neurophysiol.

[3] Roetenberg D, Buurke JH, Veltink PH, Forner Cordero A, Hermens HJ (2003). Surface electromyography analysis for variable gait. Gait Posture..

[4] Ivanenko YP, Dominici N, Cappellini G, Lacquaniti F (2005). Kinematics in newly walking toddlers does not depend upon postural stability. J Neurophysiol.

[5] Di Giulio I, Maganaris CN, Baltzopoulos V, Loram ID (2009). The proprioceptive and agonist roles of gastrocnemius, soleus and tibialis anterior muscles in maintaining human upright posture. J Physiol.

[6] Balaban B, Tok F (2014). Gait disturbances in patients with stroke. PM R.

[7] Davids JR, Rogozinski BM, Hardin JW, Davis RB (2011). Ankle dorsiflexor function after plantar flexor surgery in children with cerebral palsy. J Bone Joint Surg Am.

[8] Khanmohammadi R, Talebian S, Hadian MR, Olyaei G, Bagheri H (2016). Characteristic muscle activity patterns during gait initiation in the healthy younger and older adults. Gait Posture.

[9] Farina D, Merletti R, Nazzaro M, Caruso I (2001). Effect of joint angle on EMG variables in leg and thigh muscles. IEEE Eng Med Biol Mag.

[10] Rainoldi A, Melchiorri G, Caruso I (2004). A method for positioning electrodes during surface EMG recordings in lower limb muscles. J Neurosci Methods.

[11] Minetto MA, Botter A, Šprager S, Agosti F, Patrizi A, Lanfranco F (2013). Feasibility study of detecting surface electromyograms in severely obese patients. J Electromyogr Kinesiol.

[12] Farina D, Cescon C, Merletti R (2002). Influence of anatomical, physical, and detection-system parameters on surface EMG. Biol Cybern.

[13] Mesin L, Merletti R, Vieira TMM (2011). Insights gained into the interpretation of surface electromyograms from the gastrocnemius muscles: a simulation study. J. Biomech. Elsevier.

[14] Fukunaga T, Kawakami Y, Kuno S, Funato K, Fukashiro S (1997). Muscle architecture and function in humans. J Biomech.

[15] Vieira T, Botter A, Minetto MA, Hodson-Tole EF (2015). Spatial variation of compound muscle action potentials across human gastrocnemius medialis. J Neurophysiol.

[16] Barone U, Merletti R (2013). Design of a portable, intrinsically safe multichannel acquisition system for high-resolution, real-time processing HD-sEMG. IEEE Trans Biomed Eng.

[17] Varghese A, Bianchi S (2014). Ultrasound of tibialis anterior muscle and tendon: anatomy, technique of examination, normal and pathologic appearance. J Ultrasound.

[18] Chleboun GS, France AR, Crill MT, Braddock HK, Howell JN (2001). In vivo measurement of fascicle length and pennation angle of the human biceps femoris muscle. Cells Tissues Organs.

[19] Maganaris CN, Baltzopoulos V (1999). Predictability of in vivo changes in pennation angle of human tibialis anterior muscle from rest to maximum isometric dorsiflexion. Eur J Appl Physiol Occup Physiol.

[20] Bisi MC, Botter A, Stagni R, Vieira T. New frontiers for muscle function investigation: Integration of surface EMG and 3D ecographic images. J Mech Med Biol. 2015;15(2):1540029. doi:10.1142/S0219519415400291.

[21] Stagni R, Fantozzi S, Cappello A, Camomilla V (2006). Ultrasound for identification of anatomical landmarks in stereophotogrammetry: a new method for the calibration of the probe. J Biomech.

[22] Cappozzo A, Catani F, Croce UD, Leardini A (1995). Position and orientation in space of bones during movement: anatomical frame definition and determination. Clin Biomech (Bristol, Avon).

[23] Vieira TMM, Merletti R, Mesin L (2010). Automatic segmentation of surface EMG images: improving the estimation of neuromuscular activity. J Biomech Elsevier.

[24] Hodson-Tole EF, Loram ID, Vieira TMM (2013). Myoelectric activity along human gastrocnemius medialis: different spatial distributions of postural and electrically elicited surface potentials. J Electromyogr Kinesiol.

[25] Hermens HJ (2000). Development of recommendations for Semg sensors and sensor placement procedures. Pdf.

[26] Ruiz Muñoz M, González-Sánchez M, Cuesta-Vargas AI (2015). Tibialis anterior analysis from functional and architectural perspective during isometric foot dorsiflexion: a cross-sectional study of repeated measures. J Foot Ankle Res.

[27] Wu G, Cavanagh PR (1995). ISB recommendations for standardization in the reporting of kinematic data. J Biomech.

[28] Grood ES, Suntay WJ (1983). A joint coordinate system for the clinical description of three-dimensional motions: application to the knee. J Biomech Eng.

[29] Dietz V, Müller R, Colombo G (2002). Locomotor activity in spinal man: significance of afferent input from joint and load receptors. Brain.

[30] De Luca CJ, Merletti R (1988). Surface myoelectric signal cross-talk among muscles of the leg. Electroencephalogr Clin Neurophysiol.

[31] Chleboun GS, Busic AB, Graham KK, Stuckey HA (2007). Fascicle length change of the human tibialis anterior and vastus lateralis during walking. J Orthop Sports Phys Ther.

[32] Stegeman DF, Dumitru D, King JC, Roeleveld K (1997). Near- and far-fields: source characteristics and the conducting medium in neurophysiology. J Clin Neurophysiol.

